# Isolation and Characterization of an Antibacterial Peptide Fraction from the Pepsin Hydrolysate of Half-Fin Anchovy (*Setipinna taty*)

**DOI:** 10.3390/molecules17032980

**Published:** 2012-03-09

**Authors:** Ru Song, Rong-Bian Wei, Hong-Yu Luo, Dong-Feng Wang

**Affiliations:** 1 Department of Food Science, College of Food Science and Pharmacy, Zhejiang Ocean University, Zhoushan 316000, China; Email: windswingcn@yahoo.com.cn (R.-B.W.); hongyuluo68@yahoo.cn (H.-Y.L.); 2 College of Food Science and Engineering, Ocean University of China, Qingdao 266003, China; Email: wangfengd60@yahoo.cn

**Keywords:** half-fin anchovy pepsin hydrolysate, isolation, peptide sequence, secondary structure prediction, membrane disruption

## Abstract

Enzymatic proteolysis of food proteins is considered a promising method to generate antibacterial peptides. The objective of the present study was to isolate and characterize peptide fraction from the pepsin hydrolysate of half-fin anchovy (*Setipinna taty*) with antibacterial activity against *Escherichia coli*. The most active peptide fraction HAHp2-3-I was isolated by a series of chromatographic methods, including Sephadex G-25 chromatography, reverse high-performance liquid chromatography (RP-HPLC) and Source 5RPC ST. Peptides identification of HAHp2-3-I was carried out using UPLC-LTQ-Orbitrap mass spectrometer. HAHp2-3-I contained five cationic peptides (MLTTPPHAKYVLQW, SHAATKAPPKNGNY, PTAGVANALQHA, QLGTHSAQPVPF and VNVDERWRKL) and three anionic peptides (LATVSVGAVELCY, NPEFLASGDHLDNLQ and PEVVYECLHW). Prediction of peptide secondary structure indicated that these anionic peptides should have extended strand and random coil structures, whereas cationic peptides PTAGVANALQHA and VNVDERWRKL could form alpha helixes. In addition, results of scanning electron microscopy (SEM) revealed that treatment by HAHp2-3-I could cause the morphological changes of *E. coli* and destruction of the cell integrity via irreversible membrane damage. The results could provide information for investigating the antibacterial model of antibacterial peptides derived from fish protein hydrolysates.

## 1. Introduction

In the last two decades, the increase of bacterial resistance to commercial antibiotics and the increased restriction on the use of chemical preservatives in foods have greatly stimulated the search for novel alternative natural antimicrobial agents that possess a broad spectrum of antibiotic activity. Since the first antimicrobial peptide was identified from the cecropia moth (*Hyatophora cecropia*) [1], a variety of antimicrobial peptides (AMPs) have been found with numerous origins, such as plants, animals and microorganisms [2,3,4]. Proteolytic digests of proteins also generate AMPs, in fact, many AMPs including casecidins, isracidin and casocidin-I were initially isolated from bovine casein hydrolysates [5,6,7]. Although AMPs are mainly generated from milk source protein hydrolysates, other types of proteins, after hydrolysis, have the potential of releasing novel AMPs or enhancing bioactivities, compared with their intact forms [8,9,10]. Therefore, digestion of proteins to release AMPs might open a new way to produce antimicrobial ingredients in large scale [11].

Fish possess a strong innate immune system, which acts as the first line of defense against pathogen infections [12]. Fish AMPs and their possible applications are reported in recent papers [4,13]. These fish origin AMPs have positive charge and amphipathic characteristics like other AMPs from terrestrial animals [4]. Researchers have focused on preparation of fish AMPs from fish secretes, organ extractions, or transgenic expression [14,15,16]. However, there are few investigations on AMPs from fish hydrolysates. Half-fin anchovy (*Setipinna taty*) is an under-utilized marine fish found in the coastal waters of China. In our previous study, we found that the pepsin hydrolysate of half-fin anchovy (HAHp) possessed antibacterial activity against Gram-positive and Gram-negative bacteria [17]. The main objective of this study is to isolate and characterize the antibacterial peptide fraction from HAHp. Moreover, we discuss the possible antibacterial model, aiming at providing information for further investigation of antibacterial mechanism of fish source AMPs derived from fish hydrolysates.

## 2. Results and Discussion

### 2.1. Isolation and Identification of Antibacterial Fraction from HAHp

#### 2.1.1. Gel Permeation Chromatography

**Figure 1:**
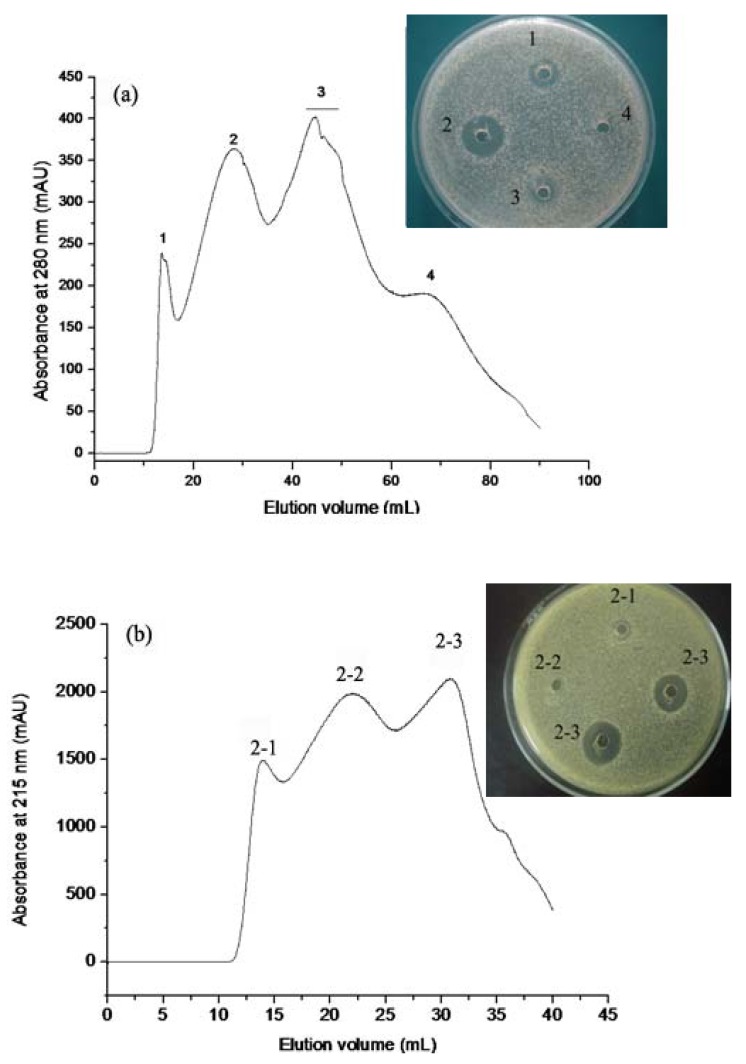
Chromatogram profiles and antibacterial activity of fractions isolated by Sephadex G-25 gel. (**a**) Eluted with 50 mM sodium phosphate buffer in 0.15 mol/L NaCl (pH 5.8) at a flow rate of 1.0 mL/min. (**b**) Eluted with deionized water at a flow rate of 0.5 mL/min.Numbers beside the wells were corresponding to the peak numbers in chromatographic diagram.

HAHp was first separated into four chromatographic peaks by Sephadex G-25 chromatography (Figure 1a). The fraction HAHp2 (represented in peak 2) showed the strongest activity against *E. coli* among all fractions attained. Bioassay guided chromatography for further separation of the fraction HAHp2 provided three additional peaks, which were eluted with deionized water on the Sephadex G-25 column. Peak 2-3 (named as HAHp2-3) demonstrated antibacterial activity on *E. coli* (Figure 1b). Peptides with net charges or hydrophobic characteristics are easily absorbed to the gel when deionized water is used as eluent, resulting in delayed peaks.

Highly cationic antibacterial peptides are easily attracted to the anionic charges that exist on the outer envelope of the cell wall of Gram-negative bacteria. Furthermore, the hydrophobic properties of peptides enable them to perturb the cell membrane integrity or translocate into the cytoplasm [18,19,20]. In this sense, the strongest antibacterial activity of HAHp2-3 of all three peaks (the others are HAHp2-1 and HAHp2-2) may be due to its net charges or hydrophobic properties. Our following analysis of the physicochemical properties of peptides confirmed this speculation.

#### 2.1.2. RP-HPLC and Source 5RPC ST purification

**Figure 2:**
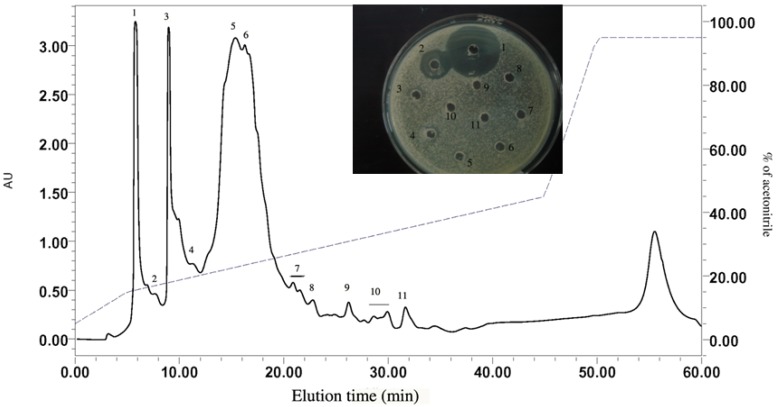
Fractionation and antibacterial activity of HAHp2-3 using preparative RP-HPLC. Numbers beside the wells were consistent with the peak numbers in RP-HPLC profile.

**Figure 3:**
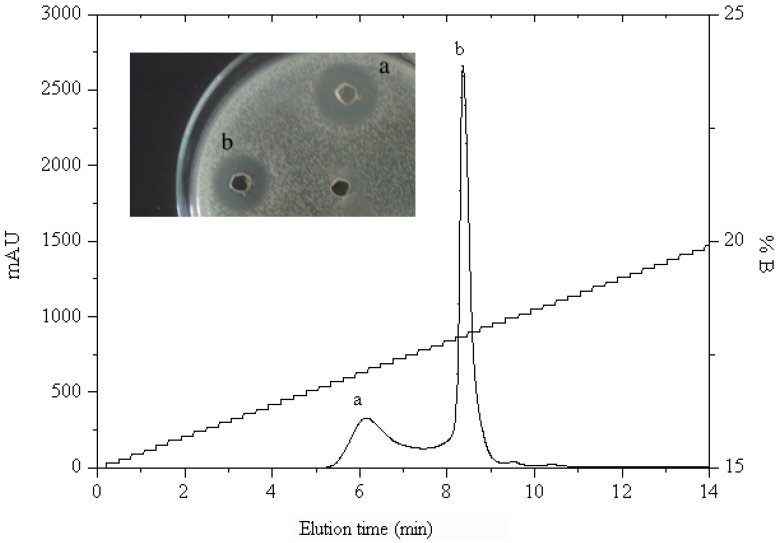
Chromatogram profile and antibacterial activity of HAHp2-3-I analyzed by Source 5RPC ST column. Numbers beside the wells represented the peak numbers in the chromatogram profile.

HAHp2-3 was further separated into several peaks by preparative RP-HPLC. The resulting peak 1 displayed the strongest antibacterial activity and was named as HAHp2-3-I (Figure 2). It was sub-fractionated into two more peaks (peak a and peak b) when analyzed with a Source 5RPC ST analytical HPLC column (Figure 3). Both peaks showed antibacterial activity. Therefore, we loaded HAHp2-3-I onto UPLC-LTQ-Orbitrap mass spectrometer for further purification and identification, aiming at revealing all peptide sequences in a simple and economical way.

### 2.2. Peptide Sequence Analysis and Physicochemical Properties

**Figure 4:**
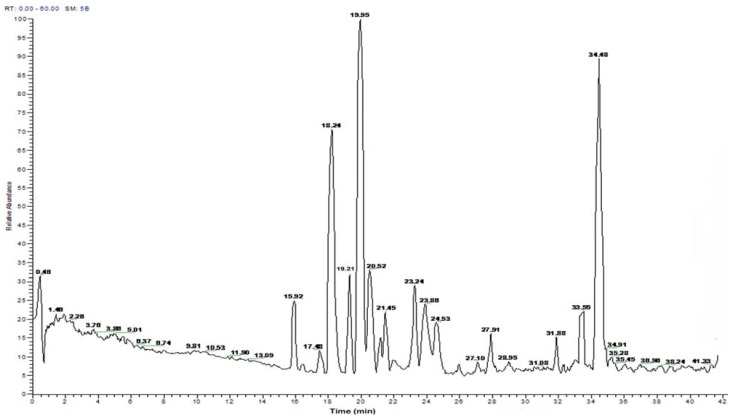
UPLC chromatogram profile (Michrom C_18_, 0.1 × 150 mm) of HAHp2-3-I. 0–5min, 5% solvent B; 5–12min, 5–8% solvent B; 12–22 min, 8–13% solvent B; 22–42min, 13–30% solvent B.

HAHp2-3-I was composed of more than ten peptides (Figure 4). Ten peptide sequences were selectively analyzed for those with eluting time of 15.92, 18.24, 19.21, 19.95, 20.52, 21.45, 23.24, 23.86, 33.55 and 34.48 min, respectively (Table 1). The molecular weights of these peptides ranged from 1,100 to 1,700 Da. The observed masses were all less than 18 Da compared to their calculated masses, which may be due to the loss of one water molecule. Eight peptides with charge were identified, including five cationic ones: MLTTPPHAKYVLQW (HAHp-cationic1), SHAATKAPPKN GNY (HAHp-cationic2), PTAGVANALQHA (HAHp-cationic3), QLGTHSAQPVPF (HAHp-cationic4), and VNVDERWRKL (HAHp-cationic5) and three anionic ones: LATVSVGAVELCY (HAHp-anionic1), NPEFLASGDHLDNLQ (HAHp-anionic2), and PEVVYECLHW (HAHp-anionic3). HAHp-catoinic2 possessed the highest cationic charges (+3), whereas its hydrophobic ratio (21%) was the lowest among the five cationic peptides. In contrast, HAHp-cationic3 had the highest hydrophobic ratio (50%) and the lowest cationic charges (+1). HAHp-catoinic1 had intermediate net charges (+2) and hydrophobicity ratio (42%). The antibacterial activity of cationic peptides can be modulated through modification of net charge or the ratio of hydrophobicity [21,22]. Nevertheless, the exposed basic residues of anionic peptides are not essential for their antibacterial activities. For example, the antibacterial activity of anionic peptides, Cn-AMP2 (TESYFVFSVGM) and Cn-AMP3 (YCSYTMEA) are related with their hydrophobic amino acid residues [19]. In this study, HAHp-anionic1 and HAHp-anionic3 displayed higher hydrophobic percentages than other peptides. Results in Table 1 indicate that the antibacterial activity of HAHp2-3-I may be due to its cationic and, or hydrophobic characteristics.

**Table 1:** Amino acid sequences of selected peptides in fraction HAHp2-3-I.

Time	Amino acid sequence	Calculated mass	Net	Hydrophobic ratio ^b^ (%)
(min)		/observed mass	charge ^a^	
15.92	MLTTPPHAKYVLQW	1685.0/1665.92	+2	42
18.24	LRSKAAAPAEQYE	1433.5/1414.78	0	38
19.21	TPGALLEHPTL	1148.3/1129.60	0	36
19.95	SHAATKAPPKNGNY	1455.5/1436.91	+3	21
20.52	LATVSVGAVELCY	1324.5/1305.73	−1	61
21.45	PTAGVANALQHA	1149.2/1130.67	+1	50
23.24	QLGTHSAQPVPF	1281.4/1262.62	+1	33
23.86	VNVDERWRKL	1314.5/1295.65	+1	40
33.55	NPEFLASGDHLDNLQ	1669.7/1650.89	−2	33
34.48	PEVVYECLHW	1274.4/1255.72	−1	50

^a^ Net charge was calculated based on negatively charged amino acid (E and D) and positively charged amino acid (K, R and H) in the peptide sequence; ^b^ Calculated the percentage of hydrophobic residues (I, V, L, F, C, M, A, W) in the peptide sequence.

### 2.3. Prediction of Peptide Secondary Structure

The ten peptides identified in fraction HAHp2-3-I all had no disulfide bridges. Peptides without disulfide bridges are in disorder when dissolved in aqueous solutions. However, when they bind to a membrane or in other hydrophobic environment, or when self-aggregation occurs, all or part of the molecule may convert to a secondary structure [23,24,25]. The predictions of secondary structure of these charged peptides derived from HAHp2-3-I are listed in Table 2.

**Table 2:** Secondary structure prediction of charged peptides in HAHp2-3-I.

Peptides	Amino acid sequence ^a^	Secondary structure ^b^
HAHp-cationic1	MLTTPPHAKYVLQW	
	ccccccccceeecc	Extended strand (21.43%), random coil (78.57%)
HAHp-cationic2	SHAATKAPPKNGNY	
	cccccccccccccc	Random coil (100%)
HAHp-cationic3	PTAGVANALQHA	
	cccchhhhhccc	Alpha helix (41.67%), random coil (58.33%)
HAHp-cationic4	QLGTHSAQPVPF	
	cccccccccccc	Random coil (100%)
HAHp-cationic5	VNVDERWRKL	
	cccchhhccc	Alpha helix (30.00%), random coil (70.00%)
HAHp-anionic1	LATVSVGAVELCY	
	ceeeeeeeeeecc	Extended strand (76.92%), random coil (23.08%)
HAHp-anionic2	NPEFLASGDHLDNLQ	
	ccceccccccccccc	Extended strand (6.67%), random coil (93.33%)
HAHp-anionic3	PEVVYECLHW	
	cceeeeeecc	Extended strand (60.00%), random coil (40.00%)

^a^ c, e and h represented secondary structures of random coil, extended strand and alpha helix, respectively; ^b^ Data in brackets represented the percentage of predicted secondary structure.

Peptides HAHp-cationic3 and HAHp-cationic5 showed the capacity of forming alpha helix structures. Moreover, the percentage of alpha helix of HAHp-cationic3 (41.67%) was obviously higher than that of HAHp-cationic5 (30.00%). HAHp-cationic1, HAHp-cationic2 and HAHp-cationic4 lacked of alpha helixes, and they were mainly or totally composed of random coils. 

As for the anionic peptides from HAHp2-3-I, they were composed of extended strand and random coil structures. Previous study showed the increased alpha helix contents for AMPs are often correlated with their stronger activities [26]. However, many AMPs which lack an alpha helix, such as indolicidin (ILPWKWPWWPWRR-NH2) and CP11CN (ILKKWPWWPWRRK-NH2) can penetrate phosphor-lipid monolayers, mediate lipid flip-flop and cause leakage of calcein across the membrane [27], whereas their secondary structures are only the random coils, as predicted by Hierarchical Neural Network tool. Our results of the prediction of peptide secondary structure shown in Table 2 are similar to that study, and concordant with the antibacterial activity exhibited by fraction HAHp2-3-I.

### 2.4. Scanning Electron Microscopy (SEM)

**Figure 5:**
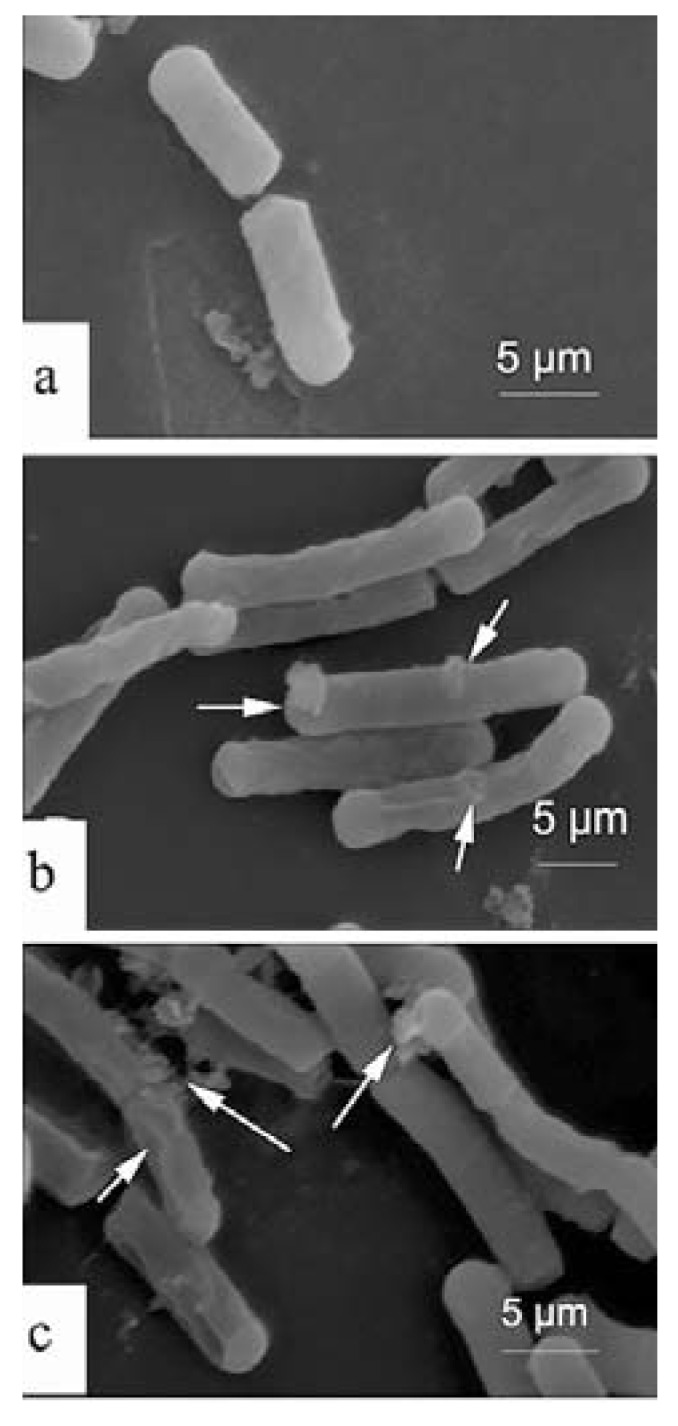
Scanning electron microscopy of *Escherichia coli* treated with HAHp2-3-I. (**a**) Control, absence of HAHp2-3-I; (**b**), (**c**) treatments, presence of HAHp2-3-I. Bars in figure represented 5 μm. Micelles, pores, and membrane disruption were marked with arrows.

In the control group a normal surface of *E. coli* can be shown and without apparent cellular debris (Figure 5a). By comparison, a variety of morphological changes were visualized in the treated groups by HAHp2-3-I (Figure 5b,c). For example, the cell membrane surface in treated groups was rougher than that of the control, with pores and micelles appearing on the membrane surfaces. In addition, membrane disruption was observed in some cells, indicating the disruption of the bilayer curvature and leakages of cellular cytoplasmic contents. The antibacterial peptides are divided into membrane disruption and non-membrane disruption mechanism classes, depending on whether the reorientation of peptide leads to perturbation of the integrity of the cell membrane or peptide translocation into the cytoplasm [18,20]. From the result of SEM, it can be inferred that HAHp2-3-I could perform antibacterial activity via membrane disruption model. 

In the membrane disruption mechanism, “barrel-stave”, “carpet” or “toroidal-pore” mechanisms are well reported. In the “barrel-stave”, or “toroidal-pore” model, it is required that the peptides span the lipid bilayer, whereas peptides with less than 20 residues are unable to span the lipid bilayer [28]. All identified peptides in HAHp2-3-I were less than 16 residues (seen in Table 1), and thus they seem unable to span the lipid bilayer through the “barrel-stave”, or “toroidal-pore” model. However, it should be mentioned that some α-helical peptides with as few as 13 residues are reported to possess antibacterial activity, so the ability to span a lipid bilayer is not a prerequisite for the antibacterial activity of peptides with α-helices [27]. In the case of the “carpet” model, antibacterial peptides with net charges will be attached to the membrane surface by electrostatic effects, and then accumulate on the bilayer surface like a carpet. Finally, the bilayer is disrupted in a detergent-like manner, and micelles are usually observed on the membrane surface [20,29,30]. In our present study, micelles and pores on the surface of *E. coli* after incubation with HAHp2-3-I were clearly observed (Figure 5b,c). In combination the physicochemical properties and prediction of peptides secondary structure (see Table 1 and Table 2) with the result of SEM, we presume that the antibacterial mechanism of HAHp2-3-I is more likely the “carpet” model. While recognition of the antibacterial mechanism of bioactive peptides is complicated and no single technique is sufficient to explain the action, the present study may, at least, provide necessary information for preparation and identification of antibacterial peptide fractions from fish hydrolysates, yet further investigations need to be done.

## 3. Experimental

### 3.1. Materials

The half-fin anchovy (*Setipinna taty*) marine fish were purchased from a local fish market in Zhoushan, China. Pepsin was provided by Sinopharm Chemical Reagent Co. Ltd (Shanghai, China). Agar, peptone and beef extract were purchased from Tianhe Microbial-agent Co. Ltd (Hangzhou, China). *Escherichia coli* strain was provided by College of Food Science and Pharmacy, Zhejiang Ocean University. Elute solutions used in the experiments were of chromatographic grade for HPLC and UPLC. Other reagents were analytical grade and commercially available.

### 3.2. Preparation of the Pepsin Hydrolysate of Half-Fin Anchovy (HAHp)

The pepsin hydrolysate of half-fin anchovy was prepared according to the optimized method described in our previous study [17]. Briefly, ground fish muscle was mixed with deionized water at a ratio of 1:4 (w/v). The pH of the mixture was adjusted to 2.0 with 6 mol/L HCl. Pepsin was added to the mixture at a ratio of 1100 U/g. The hydrolysis was performed at 37 °C for 2.4 h, terminated by heating at 95 °C for 10 min. Then the pH of the hydrolysates was adjusted to 7.0 and centrifuged at 10,000 ×*g* for 20 min (4 °C) lysate was named as HAHp. The hydrolysis degree (DH) and soluble peptides of HAHp were (18.12 ± 0.39)% and (81.78 ± 0.04)%, respectively, measured by the method described in Song *et al.* [31].

### 3.3. Antibacterial Activity against *Escherichia coli*

The agar-well diffusion method [32] with further modifications was used to determine the antibacterial activity of fractions isolated from HAHp. *E. coli* was the most susceptible bacterial strain to HAHp [17], therefore, in this study, *E. coli* was used as the indicator stain. *E. coli* suspension (100 μL) grown in a nutrient broth (18–24 h old, approx. 10^8^–10^9^ CFU/mL) was pipetted into a Petri dish, then 15 mL of nutrient agar (45–50 °C) was poured in to blend with the suspension. A sterilized steel punch was used to obtain wells (4 mm diameter) after the agar coagulated. Finally, 25 μL of sample was added to each well and incubated at 37 °C for 24 h. The formation of inhibition zone around the well was observed.

### 3.4. Isolation of Antibacterial Fraction from HAHp

HAHp was fractioned on a Sephadex G-25 column (26 × 100 mm; Amersham Pharmacia, Uppsala, Sweden) using an ÄKTA purifier system. To avoid absorption to the gel for ingredients with net charges or hydrophobic characteristics, we used 50 mM sodium phosphate buffer (pH 5.8) in 0.15 mol/L NaCl as the eluent for the first round of separation on the Sephadex G-25 column. The flow rate was 1.0 mL/min. Peak fractions were pooled and lyophilized. The fraction with antibacterial activity against *E. coli* was subject to a second chromatographic step on the Sephadex G-25 column at a flow rate of 0.5 mL/min, eluted with deionized water. The chromatographic profile was measured at 215 nm, which indicates the absorbance of peptide bonds. The bioactive peak fraction was dissolved in 5% acetonitrile containing 0.1% (v/v) TFA (solvent A), and then applied to a C_18_ preparative reversed-phase column (10 × 250 mm, 10 μm) (Sunfire^TM^, Waters, Milford. MA, USA). Solvent B was 0.1% (v/v) TFA in 95% acetonitrile. The separation was performed using a linear gradient from 15% to 45% B in 40 min at a flow rate of 2.0 mL/min, measured at 215 nm. The bioactive peak was further purified, using a Source 5RPC ST column (4.6 × 150 mm, 5 μm) (Amersham Pharmacia) in a gradient elution from 12% to 16% of 0.1% (v/v) TFA in acetonitrile in 14 min, and measured at 215 nm with a flow rate of 0.5 mL/min.

### 3.5. Peptide Sequence Analysis and Physicochemical Properties

The lyophilized antibacterial fraction from C_18_ RP-HPLC was purified by employing an UPLC Michrom C_18_ reverse phase column (0.1 × 150 mm, 200 Å, Waters) with the same buffer conditions as C_18_ RP-HPLC. The elution was carried out at a flow rate of 300 nL/min, with 5% solvent B for 5 min, 5% to 8% solvent B for 7 min, 8% to 13% solvent B for 10 min and 13% to 30% solvent B for 20 min. The separated peaks were analyzed by a LTQ-Orbitrap mass spectrometer (Thermo, Waltham, MA, USA) under ESI positive ionization mode (+2.5 Kv ionspray voltage, 200 °C capillary temperature), scanning from a mass-to-charge ratio (*m/z*) of 400–1,800. Amino acid sequences of peptides were automatically determined by ‘*de novo*’ sequence software. The physicochemical properties of peptides were predicted using web-based peptide sequence analysis tools, Expert Protein Analysis System (ExPASy) (ProtParam, http://us.expasy.org/tools/protparam.html) [33,34] and antimicrobial peptide database (APD http://aps.unmc.edu/AP/main.php).

### 3.6. Prediction of the Secondary Structure of Peptide

The secondary structures of the identified peptides were predicted by the protein sequence analysis tool of Hierarchical Neural Network in NPS@ (Network Protein Sequence@nalysis).

### 3.7. Scanning Electron Microscopy (SEM)

Exponential phase cultures of *E. coli* cells (18 h old) were incubated with the antibacterial peptide fraction HAHp2-3-I (60 µg/mL) at 37 °C for 20 h. After centrifugation for 10 min at 2000 ×*g* (4 °C), the resulting pellet was washed twice with sterile saline water, and then re-suspended in 3.0% glutaraldehyde for 12 h at ambient temperature. Subsequently, the solution was rinsed three times with 10 mM sodium phosphate buffer (pH 7.0), and dehydrated in a graded series of ethanol solutions. After critical-point drying and layering with 20 nm gold coating, the microscopy was performed with an S-3400N scanning electron microscope (Hitachi, Tokyo, Japan). Cell suspension of *E. coli* without the addition of HAHp2-3-I was treated as the control.

## 4. Conclusions

The pepsin hydrolysate of the marine fish half-fin anchovy contained antibacterial peptide fractions. HAHp2-3-I, an antibacterial peptide fraction whose molecular weight ranges from 1,100 to 1,700 Da was isolated and characterized. Peptides sequences prediction showed that HAHp2-3-I contained net charged peptides, which could form extended strands, random coils and alpha helix structures. HAHp2-3-I might exert its antibacterial activity via a membrane disruptive model in the “carpet” model way. In sum, the results of this study may provide useful information for investigation of antibacterial peptides derived from fish hydrolysates.
